# Exercise therapy to prevent and treat Alzheimer’s disease

**DOI:** 10.3389/fnagi.2023.1243869

**Published:** 2023-08-04

**Authors:** Hamed Alizadeh Pahlavani

**Affiliations:** Department of Physical Education, Farhangian University, Tehran, Iran

**Keywords:** exercise, Alzheimer’s disease, aerobic training, anaerobic training, strength training

## Abstract

Alzheimer’s disease (AD) is a progressive neurodegenerative disease in the elderly with dementia, memory loss, and severe cognitive impairment that imposes high medical costs on individuals. The causes of AD include increased deposition of amyloid beta (Aβ) and phosphorylated tau, age, mitochondrial defects, increased neuroinflammation, decreased synaptic connections, and decreased nerve growth factors (NGF). While in animals moderate-intensity exercise restores hippocampal and amygdala memory through increased levels of p-AKT, p-TrkB, and p-PKC and decreased levels of Aβ, tau phosphorylation, and amyloid precursor proteins (APP) in AD. Aerobic exercise (with an intensity of 50–75% of VO2 max) prevents hippocampal volume reduction, spatial memory reduction, and learning reduction through increasing synaptic flexibility. Exercise training induces the binding of brain-derived neurotrophic factor (BDNF) to TrkB and the binding of NGF to TrkA to induce cell survival and neuronal plasticity. After aerobic training and high-intensity interval training, the increase of VEGF, angiopoietin 1 and 2, NO, tPA, and HCAR1 in cerebral vessels causes increased blood flow and angiogenesis in the cerebellum, motor cortex, striatum, and hippocampus. In the hippocampus, exercise training decreases mitochondrial fragmentation, DRP1, and FIS1, improving OPA1, MFN1, MFN2, and mitochondrial morphology. In humans, acute exercise as an anti-inflammatory condition causes an acute increase in IL-6 and an increase in anti-inflammatory factors such as IL-1RA and IL-10. Moderate-intensity exercise also inhibits inflammatory markers such as IFN-γ, IL-1β, IL-6, CRP, TNF-α, sTNFR1, COX-2, and NF-κB. Aerobic exercise significantly increases plasma levels of BDNF, nerve growth factor, synaptic plasticity, motor activity, spatial memory, and exploratory behavior in AD subjects. Irisin is a myokine released from skeletal muscle during exercise and protects the hippocampus by suppressing Aβ accumulation and promoting hippocampal proliferation through STAT3 signaling. Therefore, combined exercise training such as aerobic training, strength training, balance and coordination training, and cognitive and social activities seems to provide important benefits for people with AD.

## Introduction

Alzheimer’s disease (AD) is a chronic neurological disorder in the elderly and the most common cause of dementia with progressive memory loss and severe cognitive decline (Liu et al., 2013). AD accounts for approximately 60–80% of dementia cases. This disease is the main cause of dementia at aging and is associated with increased adverse outcomes and mortality in the elderly (Cass, 2017). The AD Association states that approximately 33% of adults over the age of 65 will die of AD or dementia (Barnes, 2015). Therefore, AD is a major health challenge in aging, and 81% of people with AD are 75 years or older. By 2050, it is estimated that a new person will be diagnosed with AD every 33 s, amounting to nearly 1 million new cases per year (Alzheimer’s Association, 2015). With the increase in life expectancy in the world, about 46.8 million people live with dementia and it is estimated that this number will reach 131.5 million people in 2050. AD imposes one of the largest treatment costs on healthcare systems worldwide (Ryan and Kelly, 2016). Common symptoms in people with AD are weakness in retaining new information, memory loss (especially short-term memory), problem-solving problems, disorientation, and mood and personality disorders. However, the level of these changes varies among people (Cass, 2017). The causes of AD include Increased deposition of amyloid beta and phosphorylated tau, plaques caused by amyloid β-protein (Aβ) deposition, age, a genetic mutation in amyloid precursor proteins (APP), family history, social and cognitive involvement, decreased neural synaptic connections, and traumatic brain injury (Kumar and Singh, 2015). In addition, the pathogenesis of AD appears to involve hippocampal degeneration or atrophy, microglial activation, neuroinflammation, energy failure in the brain, and neuronal apoptosis (Jin et al., 2018). On the other hand, studies have reported modifiable risk factors for AD, including diabetes, high blood pressure, obesity, smoking, depression, and inactivity. Physical activity is the most effective measure to fight this disease because it seems that physical activity can indirectly change other factors of the disease (Barnes and Yaffe, 2011). A meta-analysis states that exercise reduces the risk of dementia and AD by 28 and 45%, respectively, and that higher levels of daily exercise are associated with a lower risk of AD (Lin et al., 2018). In addition, new studies show that inactivity is one of the most common risk factors for people with AD (Cass, 2017). Exercise appears to increase cerebral blood flow, increase the volume of mitochondria in the brain, increase the volume of the hippocampus, and improve neurogenesis. Therefore, exercise may improve cognitive function, reduce neuropsychiatric symptoms, and Improve daily life activities in people with AD (Cass, 2017). Exercise has also been reported to have fewer side effects and better adherence compared to medication. Therefore, exercise seems to be a promising non-pharmacological option to delay the onset of AD or slow down this disease (Ryan and Kelly, 2016). Since the rapid aging of the population and AD is a major public health problem, a safe and effective therapeutic strategy for the prevention and treatment of AD is important. Therefore, this study aims to comprehensively look at the effects of exercise on improving AD. Because the physiological benefits of exercise in AD patients are sporadic. In this study, we examine the effect of exercise on the hippocampus, synapse connections, brain blood vessels, neuron mitochondria, neuroinflammation, growth factors, and brain metabolism. Furthermore, we aim to find the appropriate duration, intensity, and type of exercise protocols for the maximal therapeutic effects of exercise in AD.

## The effect of exercise on the hippocampus and amygdala in Alzheimer’s disease

**FIGURE 1:**
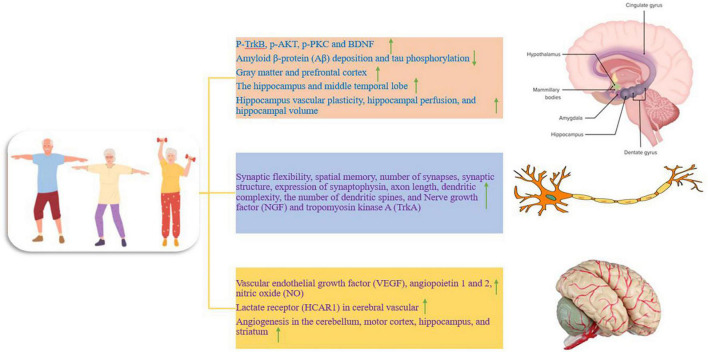
The effect of training on different parts of the nervous system, which is divided into three separate parts. Up and down arrows mean increasing and decreasing the desired factor.

Alzheimer’s disease (AD) is a progressive neurological disease with learning and memory impairment (Liu et al., 2011). The hippocampus is one of the brain regions for neurogenesis throughout life, which is critical for normal learning and memory (Ryan and Kelly, 2016). Imaging studies show neurodegeneration in the hippocampus and amygdala in the early stages of AD. Before amyloid deposition, amygdala-related long-term memory is impaired in AD mice. Thus, in mice neuronal dysfunction in the hippocampus and amygdala is evident before the onset of amyloid deposition. In dendritic complexes of basolateral amygdalar neurons, the level of signaling molecules p-TrkB, p-AKT, and p-PKC in the amygdala and hippocampus are reduced. In addition, the concentration of Aβ40 and Aβ42 is higher in the amygdala than in the hippocampus. Neurodegeneration, reduced learning performance, reduced dendritic tree complexity, and impaired brain-derived neurotrophic factor (BDNF) signaling are more serious in the amygdala than in the hippocampus. Increased soluble Aβ40 and Aβ42 in the amygdala may be the cause of neurodegeneration. In AD, the deposition of amyloid plaques in the hippocampus and amygdala is more than in normal people (Lin et al., 2015). While moderate-intensity exercise appears to enhance neural function associated with the hippocampus and amygdala. Treadmill training (ten weeks) increases memory related to the hippocampus and dendritic tree of CA1 and CA3 neurons and restores memory related to the amygdala and dendritic tree of basolateral amygdalar neurons in Alzheimer’s mice. Similarly, exercise also increases the levels of p-TrkB, p-AKT, and p-PKC in the hippocampus and amygdala (Figure 1). In addition, exercise reduces the level of soluble Aβ in the amygdala and hippocampus. In conclusion, exercise protects the amygdala and hippocampal neurons from Alzheimer’s-related degeneration, possibly through improving BDNF signaling pathways and Aβ clearance (Lin et al., 2015). The beneficial effect of running appears to be enhanced by BDNF/TrkB signaling because BDNF is an important regulator of synaptic plasticity (Liu et al., 2011). On the other hand, running (with a speed of 5 to 11 meters per minute for 30 min and 5 days a week) on a treadmill for 5 months leads to a severe reduction of Aβ deposition and tau phosphorylation in the hippocampus of Alzheimer’s mice (Figure 1). This process is associated with a significant decrease in the phosphorylation of the APP and the expression of Presenilin 1. In addition, exercise reduces Alzheimer-like neurodegeneration through the inactivation of the glycogen synthase kinase 3 (GSK3) signaling pathway. Because increased GSK3 activation appears to be associated with increased APP, Aβ production, and tau phosphorylation. Therefore, exercise is sufficient to inhibit the progression of Alzheimer’s-like neurological damage in the hippocampus and probably regulates the processing of APP in favor of reducing Aβ deposition. Because exercise prevents the accumulation of Aβ plaques and the production of Aβ in the hippocampus of mice with AD (Liu et al., 2013). In an Alzheimer’s model, exercise on a treadmill (at a speed of 10–15 m/min for 4 weeks) prevents the increase of APP and Aβ in the hippocampus. Therefore, exercise may change the balance of the APP pathway to a non-pathogenic side by increasing BDNF in the brain (Alkadhi and Dao, 2018). There is also evidence that the voluntary exercise group (16 weeks of wheel running, for 1 h every day, 5 days a week) had fewer plaques than the sedentary groups, while the forced running group (16 weeks of treadmill running) had a moderate number of plaques compared to the voluntary group and sedentary animals. Voluntary and forced exercise groups have larger hippocampal volumes than sedentary animals. In other words, voluntary exercise is probably better for reducing Alzheimer’s symptoms (such as plaque deposition and memory impairment) than forced exercise (Yuede et al., 2009). A controlled trial compared aerobic exercise 150 min per week (26 weeks) with anaerobic exercise in people with AD (mean age 72.9 years). An increase in functional ability was reported in the aerobic group compared to the anaerobic group. Therefore, it seems that cardiorespiratory fitness has a positive relationship with memory performance and bilateral hippocampus volume (Morris et al., 2017). Therefore, exercise may act as a medicine to delay the onset of Alzheimer’s (Lin et al., 2015).

## The effect of exercise on total brain volume and hippocampus in Alzheimer’s patients

Total brain volume and hippocampus differ between healthy adults and patients with dementia. During aging, brain volume and gray matter volume decrease, and higher cardiorespiratory fitness is associated with less reduction of gray matter and prefrontal cortex in elderly people (Figure 1; Barnes, 2015). In other words, a larger brain volume may lead to more storage or a higher baseline (Barnes, 2015). In addition, the hippocampus, along with the medial temporal lobe and its subcortical circuits, is compromised in early aging and under neurodegenerative conditions, particularly in AD. Hippocampal circuits are critical for episodic memory or the ability to remember unique events and experiences. Shrinking of the hippocampus in late adulthood has been reported to lead to increased memory impairment and increased dementia (Duzel et al., 2016). Therefore, AD is characterized by deficits in learning and memory, and sedentary mice show impairment in spatial memory (Mu et al., 2022). While preliminary data in humans and animals suggest that improvements in brain function require 3 to 6 months of moderate-to-high-intensity exercise (Duzel et al., 2016). There is evidence that 12 weeks of treadmill training (for 1 h per day, 5 days per week, with the intensity of exercise 50 to 65% of the maximum oxygen consumption) leads to increased synaptic flexibility, increased spatial memory, increased number of synapses, increased synaptic structure, increased expression of synaptophysin (presynaptic marker), increased axon length, increased dendritic complexity, and the increased number of dendritic spines in mice with AD (Figure 1). Therefore, exercise training through enhancing synaptic plasticity represents a potential mechanism to prevent the reduction of spatial learning and memory and the loss of synapses in mice with AD (Mu et al., 2022). In addition, the volume of the anterior hippocampus and middle temporal lobe is greater in elderly people with higher aerobic capacity (walking for 40 min with a heart rate of 50–75%) and physical activity training increases hippocampal perfusion and improves spatial memory (Figure 1). Increased hippocampal volume is associated with higher serum levels of BDNF as a mediator of neurogenesis in the dentate gyrus. Therefore, aerobic exercise training is effective in reversing hippocampal volume reduction in late adulthood, which is associated with improved memory performance (Erickson et al., 2011). Therefore, exercise even if started after middle age can have protective effects because it prevents the decline of neuroplasticity and preserves memory function. Exercise exerts protective effects against synaptic toxicity of Aβ in the hippocampus and improves Aβ degradation and clearance. In humans, normal levels of exercise are associated with reduced brain Aβ, reduced insulin, and reduced triglycerides (Duzel et al., 2016). Aerobic exercise (30-min interval training, three times a week for 12 weeks at an exercise intensity of 65% heart rate) induces vascular plasticity in the hippocampus and is associated with changes in hippocampal perfusion and hippocampal volume, which in older humans (60–77 years) decreases (Figure 1). These findings show the preservation of the vascular capacity of the aging human hippocampus caused by exercise, which decreases with age (Maaß et al., 2015). Nerve growth factor (NGF) also plays a role in promoting neuronal function and survival of neural progenitors, and hippocampal NGF expression increases after running at 2–3 days post-exercise (Neeper et al., 1996). Furthermore, hippocampal NGF and its receptor tropomyosin kinase A (TrkA) are increased after 8 weeks of running in rodents. These exercises cause the binding of BDNF to TrkB, and the binding of NGF to TrkA to stimulate cyclic AMP response element (CRE)-binding protein (CREB) and induce cell survival and neural plasticity (Figure 1; Lin et al., 2018). In this regard, cycling with moderate-intensity (20–50 min per session, 3 times a week and for 6 months) has been introduced as a potential treatment to increase the quality of life, which reduces the high costs of the dementia population (Yu et al., 2014). Therefore, exercise consistently enhances brain function by inducing structural and neurochemical changes in the hippocampus and temporal lobe circuits that are important for learning and memory (Erickson et al., 2011). Collectively, these studies highlight the role of regular exercise in maintaining brain volume in old age as a preventive tool against AD and dementia (Barnes, 2015). However, there are few reports that show that aerobic and resistance exercise (at least 300 min per week for 6 to 18 months) does not lead to significant improvement of episodic memory and cognitive disorders in elderly people. It is worth mentioning that this study used healthy elderly people with university education and no cognitive impairment, and it seems that the severity and duration of the disease and the stage of the disease can be important in the treatment results (Lenze et al., 2022).

## Exercise and increased vascularity in Alzheimer’s disease

Studies point to the role of vascular dysfunction in the pathophysiology of AD clearly and consistently. In AD, there is an increased risk for hypertension, diabetes, increased homocysteine, and their accumulation. In middle age, chronic vascular overload seems to promote AD symptoms and progression in vulnerable individuals (Lange-Asschenfeldt and Kojda, 2008). Brain blood flow undergoes unfavorable changes in old age and is associated with a lack of cognitive ability (Barnes, 2015). Hence, prolonged vascular protective actions through angiogenesis may positively influence the onset of AD (Lange-Asschenfeldt and Kojda, 2008). It has been reported that moderate-intensity exercise leads to an acute increase in blood flow to the brain, and there is an acute increase in blood flow to the brain in trained men compared to sedentary individuals (Barnes, 2015). A trial of exercise training over 12 weeks showed that resting cerebral blood flow increased in the anterior part of the brain (Barnes, 2015). In 22-month-old mice, after training (treadmill running for 3 weeks every day for 30 min), an increase in vascular endothelial growth factor (VEGF), angiopoietin 1 and 2, as well as the density of small vessels in the brain has been reported (Figure 1). This process is accompanied by a significant increase in angiopoietin 1 and 2 mRNA expression in the group of aged rats. Also, a significant increase in four VEGF mRNA isoforms (120, 144, 164, 188) has been observed. In addition, the expression of VEGF protein is also significantly increased. Therefore, it seems that angiogenesis can be enhanced in old mice through exercise, and the angiogenesis response to chronic physical activity is preserved with aging (Ding et al., 2006). High-intensity interval exercise (5 days per week for 7 weeks) can improve brain function through lactate receptors (HCAR1) in cerebral vascular cells and intracerebral microvessels (Figure 1).

HCAR1 activation also increases VEGFA and brain angiogenesis. Also, subcutaneous injection of L-lactate, similar to the increase in blood lactate levels during exercise, increases brain VEGFA protein and capillary density, but not in HCAR1-deficient mice. Therefore, exercise-induced lactate is detected through a brain receptor and produces angiogenic effects in the brain. A possible mechanism of lactate is the phosphorylation of ERK1/2 as well as Akt in the hippocampus of wild-type mice. Thus, Akt and ERK1/2 are regulators of HCAR1 on VEGFA and angiogenesis in the hippocampus (Morland et al., 2017). In animal models, exercise increases VEGF and subsequently enhances neurogenesis and angiogenesis in the brain, which is useful for reducing AD and depression-like behaviors. VEGF in the brain appears to have beneficial therapeutic effects against neurological diseases (Echeverria et al., 2017). Two-month-old rats with neurological disorders through voluntary exercise improve spatial memory impairment by increasing angiogenesis, reducing oxidative stress, and reducing Aβ neurotoxicity (Wang et al., 2013). In humans, aerobic exercise (30 min of moderate-intensity exercise at 65–75% of target heart rate reserve) significantly increases the serum levels of BDNF, IGF-1, and VEGF in elderly people with mild cognitive impairment. While acute resistance exercise (30 min with a moderate-intensity of 75% of one maximum repetition on bodybuilding machines and free weights) only increases serum IGF-1 levels. However, these factors return to baseline levels approximately 20 min after acute exercise (Tsai et al., 2018). In mice, aerobic exercise (30 days of running) induces angiogenesis and increases blood flow in the cerebellum, motor cortex, and hippocampus, and these effects seem to be sustained in the long term (Figure 1). An increase in microvessel density in the striatum after exercise has been demonstrated because angiogenic factors are stimulated and increased after 1–3 weeks of exercise, and angiogenesis increases after 3 weeks. It has been reported that older adults undergoing regular exercise have more stable cerebral blood flow compared to inactive controls (Hooghiemstra et al., 2012). Exercise also increases cerebral blood supply through nitric oxide (NO) and tissue plasminogen activator (tPA) (Figure 1). While the reduction of blood supply and metabolism in the brain is one of the causes of AD and contributes to cognitive decline. NO, derived from vascular endothelial nitric oxide synthase (eNOS), affects vascular tone, blood pressure, and cerebrovascular homeostasis and plays a vital role in cerebral perfusion. Brain autoregulation through NO reduces the risk of atherosclerosis (Eggermont et al., 2006). In general, exercise increases both neurogenesis and angiogenesis. Therefore, exercise may reduce the symptoms of neurological diseases such as AD by dealing with vascular stress in old age (Lange-Asschenfeldt and Kojda, 2008).

## Exercise to improve mitochondrial dysfunction in Alzheimer’s disease

**FIGURE 2:**
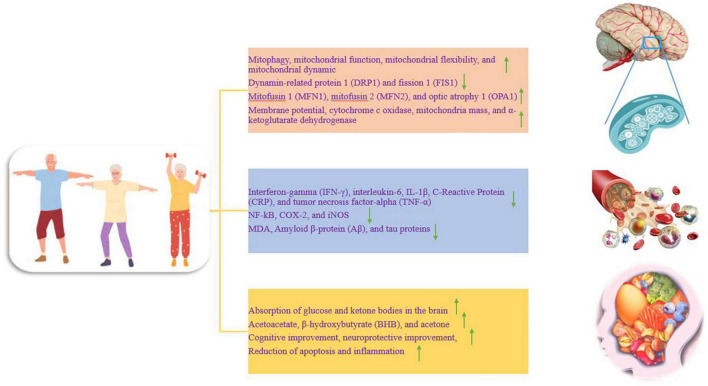
The effect of training on different parts of the nervous system, which is divided into three separate parts. Up and down arrows mean increasing and decreasing the desired factor.

Neurons are highly specialized cells and depend on mitochondria for their high energy demand (Moreira et al., 2006). Neurodegenerative disorders such as AD are associated with mitochondrial dysfunction, and the accumulation of dysfunctional mitochondria has been reported as an early stage of AD. Mitochondrial dysfunction leads to energy deficiency, calcium imbalance, oxidative stress, Aβ accumulation, increased hyperphosphorylation, cognitive decline, and memory loss (Liang et al., 2021). Mitochondrial disorder in AD is characterized by decreased activities of complex I, complex IV, complex V, pyruvate dehydrogenase complex, and alpha-ketoglutarate dehydrogenase complex and increased activation of reactive oxygen species (ROS). Brain mitochondria exposed to Aβ show decreased mitochondrial respiration in modes III and IV, decreased alpha-ketoglutarate dehydrogenase, decreased pyruvate dehydrogenase, increased ROS, mitochondrial membrane permeability, mitochondrial membrane depolarization, and a significant decrease in the adenosine triphosphate (ATP)/adenosine diphosphate (ADP) ratio. Mitochondria exposed to Aβ trigger apoptotic cascades and organelle swelling (Johri, 2021). While proper exercise has beneficial effects on improving mitophagy, and mitochondrial function, promoting mitochondrial flexibility, reducing oxidative stress, increasing cognitive capacity, and reducing cognitive disorders and dementia (Figure 2). Therefore, these factors seem to prevent the neurodegeneration process in AD through exercise (Liang et al., 2021). In the hippocampus, 12 weeks of high-intensity interval training (HIIT) and moderate-intensity continuous training (MICT) significantly decrease Aβ and mitochondrial fragmentation and improve mitochondrial morphology. In addition, both interventions downregulate fission proteins such as dynamin-related protein 1 (DRP1) and fission 1 (FIS1), while upregulating fusion proteins such as mitofusin 1 (MFN1), mitofusin 2 (MFN2), and optic atrophy 1 (OPA1) (Li et al., 2019; Figure 2). To maintain a healthy mitochondrial network, the process of fission and fusion are performed together. Fission separates dysfunctional mitochondria from healthy ones, while fusion reduces mitochondrial dysfunction by increasing network coupling and facilitating the redistribution of metabolites, proteins, and mtDNA (Alizadeh Pahlavani et al., 2022). Therefore, exercise improves the exploratory behavior, spatial learning, and memory ability of rats with AD, which is probably done by improving the morphology and dynamics of mitochondria (Li et al., 2019). In rats, offspring of sedentary mothers exposed to Aβ have learning defects and memory loss along with synaptophysin reduction, BDNF reduction, and mitochondrial dysfunction. While regular swimming of mothers during pregnancy has a neuroprotective role against Aβ in adult children by preventing the reduction of synaptophysin. The increase in functional mitochondria is accompanied by an increase in the mass of mitochondria, an increase in the membrane potential, an increase in the enzyme α-ketoglutarate dehydrogenase, and the activity of cytochrome c oxidase enzymes (Figure 2). In addition, maternal exercise during pregnancy induces long-term modulation of fusion and fission proteins such as MFN1 and DRP1 (Klein et al., 2019). Exercise significantly changes the ratio of NAD+/NADH and increases the expression of SIRT1 in the brain. Exercise appears to increase mitophagy activity by activating the SIRT1-FOXO1/3-PINK1-Parkin pathway by increasing the NAD+/NADH ratio, thus contributing to the reduction of mitochondrial dysfunction associated with AD neurodegeneration. In the hippocampus of aged rats, after treadmill exercise, PINK1-parkin-dependent mitophagy and resistance to age-related mitochondrial dysfunction are increased, and it attenuates Aβ-induced cognitive decline and mitochondrial dysfunction in AD animals. Thus, exercise exerts its therapeutic effect on AD by reversing mitochondrial dysfunction via the SIRT1-FOXO1/3-PINK1-Parkin mitophagy pathway (Zhao et al., 2021). Thus, restoring mitochondrial function with exercise can delay the onset of AD and slow the progression of the disease (Johri, 2021).

## Exercise to prevent Alzheimer’s disease through the immune system

Alzheimer’s disease (AD) is divided into two categories: early-onset AD (EOAD) and late-onset AD (LOAD), and in most cases, about 95% belong to LOAD. The most important factors for LOAD are aging, obesity, diabetes, and cardiovascular diseases. Meanwhile, the common feature of these factors is a chronic systemic inflammatory response at a low level (Scheltens et al., 2021). The brain’s immune system and inflammation in the blood greatly contribute to the development of AD due to the accumulation of tau protein, nerve damage, dementia, and cognitive impairment (Uddin et al., 2020a). With aging, the integrity and permeability of the blood-brain barrier (BBB) are lost, and bacterial metabolites and immunoglobulins enter the brain to activate microglia and the central immune system (Newcombe et al., 2018). Under normal conditions, microglia play an essential role in removing the accumulation of misfolded proteins, but during microglial dysfunction and the presence of harmful factors for a long time such as inflammation, they do not perform cleaning and monitoring, and accelerate neuroinflammation and neurodegeneration (Nichols et al., 2019). On the other hand, a variety of danger-associated molecular patterns (DAMPs) and pathogen-associated molecular patterns (PAMPs) are released to activate nervous system immune responses. PAMPs and DAMPs play a protective role to activate microglia and release pro-inflammatory cytokines. However, DAMPs and PAMPs amplify levels of chronic inflammation with aging. Despite these chronic stimuli, activated microglia become a long-term source of inflammatory factors (Chiarini et al., 2020). Activated microglia induce nuclear factor-kappa-B (NF-κB) entry into the nucleus, leading to the upregulation of inflammatory factors such as IL-1β and IL-18 to restore neuronal homeostasis. In the cerebrospinal fluid (CSF) and blood of AD patients, the levels of CD45 lymphocytes, IL-17, IFN- γ, and IL-6 are increased (Wang et al., 2023). Blocking inflammation in microglia reduces neuroinflammation and delays AD. As the most glial cells in the brain, astrocytes regulate blood flow, maintain the blood-brain barrier and create a stable environment for synapses and neurotransmitters (Lee et al., 2010). However, the secretion of inflammatory factors by active microglia such as IL-1α and TNF-α transform astrocytes into a pro-inflammatory phenotype, responsible for developing degenerative tauopathies (Kwon and Koh, 2020; Mann et al., 2022). In CSF from 300 people over the age of 60, people without dementia also show higher inflammation and increased C-reactive protein (CRP) in the blood of people with AD (Grimaldi et al., 2019). In animals, persistent interleukin 1 beta (IL-1β) expression in the hippocampus also activates astrocytes and microglia for a strong inflammatory response and subsequently causes memory impairment (Shaftel et al., 2007). The World Health Organization (WHO) recently recommends adults do 150–300 min of moderate-intensity physical activity or 75–150 min of high-intensity physical activity per week (Nieman and Wentz, 2019). The effect of exercise on inflammation depends on the type, intensity, duration of exercise, and individual or tissue differences (da Luz Scheffer and Latini, 2020). Regular moderate-intensity exercise enhances the anti-inflammatory state, but high-intensity exercise activates the inflammatory response (Pedersen and Toft, 2000; Pinho et al., 2010). Exercise releases ROS and reactive nitrogen species (RNS) by inducing an inflammatory response, muscle damage, immunosuppression, and glycogen depletion (Nieman and Wentz, 2019). Maximal inflammation in the first few hours is counteracted by anti-inflammatory effects after exercise (Pedersen and Toft, 2000). Thus, exercise increases immune system activity and reduces infection by redistributing immune cells to tissues (Campbell and Turner, 2018). Moderate-intensity exercise consistently leads to modulation of the immune system, strengthening the defense system against infection, and reducing chronic diseases (Nieman and Wentz, 2019). Therefore, the anti-inflammatory effects of exercise are more likely to be produced by long-term moderate-intensity physical activity.

## Exercise to reduce markers of inflammation in Alzheimer’s disease

There is significant inflammation in AD, and AD may be due to inflammation in the brain, and inflammation may is maintained by the Aβ protein, leading to dysfunction and loss of neurons (Kelly, 2018). Reactive oxygen species (ROS), cytokines, and markers of neuroinflammation derived from microglia and astrocytes are the dominant molecules in the inflammatory process. Neuroinflammation is related to the activation of various genes including COX-2, iNOS, nuclear factor kappa B (NF-κB), and cytokines (Leem et al., 2011). Probably, anti-inflammatory strategies such as exercise are effective treatments for AD because regular physical activity has a protective effect against AD in epidemiological studies. It seems that exercise creates an anti-inflammatory environment in the peripheral organs and brain, and by modulating neuroinflammation, it reduces cellular and cognitive damage in AD (Kelly, 2018). In humans, acute exercise is a pro-inflammatory process but is then compensated by an anti-inflammatory response. Acute exercise-induced increases in IL-6 increase plasma levels of anti-inflammatory cytokines such as IL-1RA and IL-10. IL-1RA inhibits IL-1β signal transduction, while IL-10 inhibits the production of pro-inflammatory cytokines such as TNF-α (Steensberg et al., 2003; Abd El-Kader and Al-Jiffri, 2016; Chow et al., 2022; Figure 2). For example, 16 weeks of moderate-to-high-intensity training (> 70% of maximal heart rate) prevents the development of inflammatory markers such as interferon-gamma (IFN-γ), interleukin-6, CRP, tumor necrosis factor-alpha (TNF-α), and soluble tumor necrosis factor receptor-1 (sTNFR1) (Jensen et al., 2019a; Wang et al., 2023; Figure 2). On the other hand, chronic endurance exercise [running at a speed of 12 m/min (moderate-intensity) or 19 m/min (high-intensity) for 1 h a day and 5 days a week for 3 months] decreases the expression of TNF-α, IL-6, IL-1β, COX-2, and iNOS (Figure 2). In addition, nuclear NF-kB activity is strongly decreased after exercise. Therefore, running on a treadmill for three months reduces tau phosphorylation and inflammatory markers (Leem et al., 2011). In addition, three weeks of exercise alters the innate immune response to an adaptive response and leads to a decrease in soluble Aβ, IL-1β, and TNFα in the hippocampus, a change associated with neuroprotection (Nichol et al., 2008; Figure 1).

Vitamin D and exercise (swimming for 30 min once daily for 4 weeks) can also show a significant decrease in the levels of IL-6, MDA, amyloid β, and tau proteins, and a significant increase in the levels of IL-10, dopamine, BDNF, and NGF. Therefore, it seems that the combination of vitamin D and exercise can be considered an effective strategy for the treatment of AD (Medhat et al., 2020). In addition, one month after starting the resistance exercise (RE) (5 times per week over 4 weeks), the reduction of Aβ plaques occurs, and the levels of inflammatory cytokines such as IL-1α, IL-4, and IL-6 return to normal levels. Hence, RE has critical effects on motor behavior, Aβ plaque, and inflammation in AD pathology and can be used as a non-invasive treatment to improve clinical symptoms and neurological changes in AD (Hashiguchi et al., 2020). The anti-inflammatory role of exercise appears to be related to a direct effect on immune adaptations that occur locally in skeletal muscle. Skeletal muscles release IL-6, which causes the production of anti-inflammatory cytokines, such as IL-10, and reduces pro-inflammatory cytokines, such as TNF-α and IL-1β (Kelly, 2018). Regular exercise seems to have an anti-inflammatory effect and improves brain redox, and improves the pathophysiological symptoms of AD such as Aβ deposition (Valenzuela et al., 2020). Thus, exercise to improve cognitive function in AD is associated with Aβ reduction, increased neurogenesis, or reduced inflammation. In support of this, a study with 160,000 participants stated that regular exercise reduced the risk of AD by 45% (Hamer and Chida, 2009). In general, exercise improves neuroinflammation in AD through 4 pathways. A. Exercise suppresses chronic inflammation in the body by reducing inflammatory factors and immune cells. B, exercise restores the permeability and integrity of the BBB by repairing the damage of endothelial cells and tight junctions and prevents the entry of inflammatory agents and immune cells into the brain. C. Exercise inhibits the pro-inflammatory M1 phenotype and stimulates the anti-inflammatory M2 phenotype in the brain to restore homeostasis. D, Exercise stimulates hippocampal neurogenesis in the brain by inducing BDNF expression in the brain, leading to the formation of new neurons, astrocytes, and oligodendrocytes. New cells replace old and damaged cells and regenerate the neuroinflammation. Therefore, through this process, exercise inhibits the neuroinflammatory response and delays AD, and reduces symptoms (Wang et al., 2023).

## Exercise and ketogenic diet for Alzheimer’s patients

**FIGURE 3:**
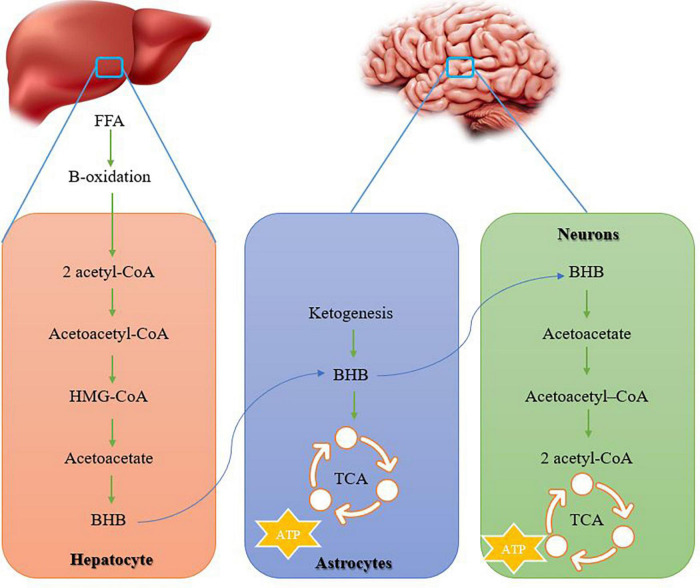
How ketone bodies are formed in the liver and transferred to the nervous system. Ketone bodies provide energy in the nervous system. acetyl-CoA, acetyl coenzyme A; HMG-CoA, 3-hydroxy-3-methylglutaryl-CoA; BHB, β-hydroxybutyrate; TCA, tricarboxylic cycle.

In AD conditions, the lack of brain glucose uptake in about 10% is caused by Presenilin-1 mutation, the presence of one or two alleles of apolipoprotein E4 (APOE4), family history of AD, insulin resistance, or age over 65 years. Therefore, the lack of brain glucose is a precursor before any cognitive deficit and probably plays a role in the deterioration of brain structure and function under AD conditions (Fortier et al., 2019). The brain under AD conditions shows reduced glucose metabolism but can use ketones for energy production. The metabolism of ketone bodies such as β-hydroxybutyrate (BHB) and acetoacetate, as physiological alternative fuels, are easily used by brain cells and do not decrease (Lange et al., 2017). Ketone bodies remain normal with normal aging and even in people with AD. It is worth noting that brain ketone level is positively correlated with blood ketone level, and interventions aimed at increasing blood ketone level should increase brain ketone level and provide more energy to compensate for brain glucose deficiency in AD (Avgerinos et al., 2020). The main site of ketone synthesis is hepatocytes, but they occur in small amounts in the kidneys and astrocytes. A large amount of acetyl coenzyme A (acetyl-CoA) is essential for ketogenesis and is mainly obtained through lipolysis of free fatty acids, while the excess production of acetyl CoA does not enter the tricarboxylic cycle (TCA) to produce ATP. In the liver, ketogenesis begins through 2 molecules of acetyl-CoA to form acetoacetyl-CoA (Figure 3). Then acetoacetyl-CoA combines with another acetyl-CoA to form 3-hydroxy-3-methylglutaryl-CoA (HMG-CoA) (Jensen et al., 2020; Figure 3). Subsequently, an acetyl-CoA is removed from HMG-CoA to produce acetoacetate, then acetoacetate can be converted to either acetone (with the lowest metabolic contribution) or BHB (the most abundant ketone body in human circulation). Acetoacetate and BHB are not used in the liver and are released into the circulation to extrahepatic tissues for metabolism (Makievskaya et al., 2023; Figure 3). In astrocytes and neurons, acetoacetate and BHB are converted to acetyl-CoA and enter the Krebs cycle and electron transport chain (ETC) to form ATP (Jensen et al., 2020; Uddin et al., 2020b; Figure 3). For example, during fasting, a change in the metabolism of glucose to ketones has been reported in the body, and acetoacetate and BHB provide almost 50% of brain energy (Cahill, 2006). The main approaches to increasing blood and brain ketones are through fasting, exercise, and ketogenic diets (restricted carbohydrates with high fat or low calories). Ketone levels can be increased by consuming medium-chain triglycerides (MCTs). A meta-analysis with 422 participants shows that compared to a placebo, MCTs increase beta-hydroxybutyrate and cause cognitive improvement in AD (Avgerinos et al., 2020). A long-term ketogenic diet with an increase in blood ketone concentration is effective in improving general cognition and episodic memory so that absorption and use of brain ketone improve (Grammatikopoulou et al., 2020). A 71-year-old woman with apolipoprotein E (ApoE4) and a family history of AD developed significant reductions in triglycerides, very low density lipoprotein (VLDL), and HgA1c after 10 weeks of a ketogenic diet combined with exercise, and increased baseline cognitive scores. Hence, a ketogenic diet and exercise seem to be beneficial for patients with AD (Morrill and Gibas, 2019). Therefore, aerobic exercise is probably useful for increasing endogenous plasma ketones through the release of free fatty acids from adipose tissue and leads to the facilitation of brain ketone absorption because 3 months of moderate-intensity aerobic exercise leads to a doubling of brain ketone absorption, while brain glucose absorption remains unchanged. In this regard, a 2-year study on the elderly recommends diet, exercise, and cognitive training to improve the cognition of the elderly (Cunnane et al., 2022).

On the other hand, moderate-intensity aerobic exercise (walking for 3 months with an average of 8 km/week and a speed of 4 km/h) in 10 patients with mild AD (with an average age of 73 years) leads to 2–3 times the absorption of glucose and acetate in the brain. Therefore, in patients with mild AD, aerobic exercise improves brain energy metabolism and cognitive disorders by increasing the absorption of ketones and glucose in the brain (Castellano et al., 2017; Figure 2). Accordingly, the brain is fed BHB and acetoacetate during starvation, fasting, and exercise. In addition, ketones play a neuroprotective role in the brain of old people by preventing memory impairment and neurodegeneration. Ketone production also improves mitochondrial function, reduces apoptosis, and reduces inflammation (Figure 2). In this regard, ketogenic nutrition combined with high-intensity interval training (HIIT) and memory training after 12 weeks leads to an improvement in neuroprotective markers (Dahlgren and Gibas, 2018; Figure 2). Thus, disruption of normal neuronal activity appears to be important for cognitive decline in AD, while the ketogenic diet acts to increase blood BHB or its downstream targets, and may have therapeutic potential in AD by improving nerve function (Le Page et al., 2017). The mechanisms of the ketogenic diet seem to be known to reduce amyloid beta and tau proteins, reduce neuroinflammation, reduce gut microbiota, reduce oxidative stress, and reduce brain metabolism (Xu et al., 2023). Therefore, the combination of ketogenic diet and exercise is recommended for AD.

## Exercise to increase BDNF in people with Alzheimer’s disease

In AD, brain neurons and synapses decrease and the patient leads to dementia, while during life, the production of new neurons in the hippocampus is called adult hippocampal neurogenesis. Adult hippocampal neurogenesis is disrupted before the onset of AD (Choi et al., 2018). Low BDNF expression predisposes neurons to oxidative stress and Aβ-induced dysfunction seen in AD (Wang and Holsinger, 2018). A decrease in BDNF levels leads to dysfunction due to a decrease in synaptic plasticity in the hippocampus and other brain regions such as the limbic system and striatum during aging (Duzel et al., 2016). In particular, BDNF is essential for neural plasticity and is highly expressed in the hippocampus, hypothalamus, and cerebral cortex. BDNF enhances long-term memory storage and reorganization of dendritic spines. BDNF action is primarily mediated by receptor tyrosine kinase B (TrkB) and initiates downstream signaling pathways for neuroprotection (Wang and Holsinger, 2018). Aerobic exercise significantly increases BDNF plasma levels in people with AD, and a significant relationship between BDNF levels and physical activity levels has been reported (Coelho et al., 2014). BDNF, nerve growth factor, glutathione, superoxide dismutase, motor activity, exploratory behavior, and spatial memory levels are lower in people with AD compared to the exercise group. But in the hippocampus, Aβ-42, tau protein, malondialdehyde, and protein carbonyl are higher (Belviranlı and Okudan, 2019). Voluntary cycling upregulates BDNF, IGF-1, and VEGF expression for neurogenesis and angiogenesis in the hippocampus and cerebral cortex. Therefore, exercise-induced upregulation of BDNF expression in hippocampal and cortical neurons may enhance cell survival and synaptic regeneration (Vivar et al., 2013). IGF-1 levels in skeletal muscle are rapidly upregulated during exercise and peak within 5–10 min. Increased circulating IGF-1 levels lead to increased IGF-1 levels in the brain because circulating IGF-1 can cross the blood-brain barrier into the brain (Vivar et al., 2013). IGF-I neutralizing antibodies block the effect of physical exercise on hippocampal BDNF expression and neurogenesis in rodents. In humans, IGF-1 is lower in inactive than in active older adults but transiently increases after exercise to significant levels in active older adults (Vivar et al., 2013). In addition, regular exercise enhances the production of neurotrophins such as BDNF by increasing metabolic factors such as ketone bodies and lactate and muscle-derived myokines such as cathepsin-B and irisin (Valenzuela et al., 2020; Alizadeh Pahlavani, 2022). Overexpression of peroxisome-activated receptor gamma coactivator-1 alpha (PGC-1α) in the muscle leads to an increase in irisin, which is released during exercise, then irisin enters the brain and induces the expression of the hippocampal BDNF gene. Therefore, the effects of exercise on cognition, adult neurogenesis, and hippocampal BDNF levels have also been reported (Valenzuela et al., 2020). Exercise-induced adult hippocampal neurogenesis improves cognition with decreased amyloid-β protein and increased BDNF, interleukin-6 (IL-6), irisin, and synaptic markers (Choi et al., 2018). Running-induced hippocampal neurogenesis is positively correlated with increased synaptic plasticity, spatial memory, and pattern separation in adult animals. Therefore, exercise can reverse neurogenesis and memory function in aged rodents (Duzel et al., 2016). Exercise seems to reduce almost all non-cognitive and cognitive impairments in AD, and increasing neurotrophic factors and ameliorating oxidative stress moderate these beneficial effects (Belviranlı and Okudan, 2019).

## Exercise-derived irisin to improve Alzheimer’s disease

**FIGURE 4:**
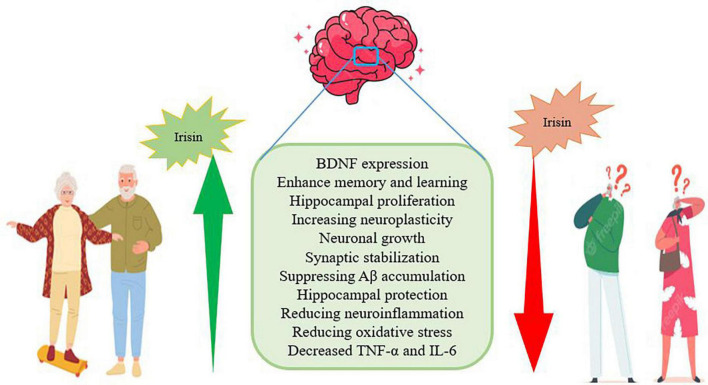
The role of exercise-induced irisin in people with AD.

Lack of physical activity is one of the factors that endanger human health and increases neurological diseases such as AD. On the other hand, exercise has many beneficial effects on brain health and cognitive function in elderly people with AD (Dehghan et al., 2021). Irisin is a myokine secreted from skeletal muscle in response to exercise and has protective functions by regulating brain-derived neurotrophic factors in the central and peripheral nervous system. Irisin is present in parts of the brain such as Purkinje cells, paraventricular nucleus, and cerebrospinal fluid (Jin et al., 2018; Young et al., 2019). In the model of rats with AD and under the injection of soluble Aβ, irisin in the hippocampus, cerebrospinal fluid is reduced. This suggests that irisin may play a role in synaptic plasticity, enhancing memory, and reducing cognitive impairment in AD (Lourenco et al., 2019; de Freitas et al., 2020). Irisin enters the central nervous system by crossing the blood-brain barrier (BBB) and increases the expression of BDNF to enhance memory and learning (Jodeiri Farshbaf and Alviña, 2021; Qi et al., 2022; Figure 4). Irisin promotes hippocampal proliferation through STAT3 signaling and may help reduce the risk of AD. This exercise-irisin-BDNF process probably increases neuroplasticity such as neuronal growth and synaptic stabilization and is a therapeutic target for AD (Moon et al., 2013; Figure 4). Irisin with a concentration of 50 and 100 nmol/liter leads to an increase in the proliferation of hippocampal cells (Moon et al., 2013). Irisin is known to protect the hippocampus, which is more vulnerable to AD, by suppressing Aβ accumulation, so it appears to be able to inhibit or delay AD (Jin et al., 2018). Irisin activates Akt and ERK1/2 signaling pathways in brain tissue, which has neuroprotective effects. In addition to skeletal muscle, irisin is also expressed in the brain, leading to reduced cerebral infarct volume, neuroinflammation, and oxidative stress reduction. Also, the plasma irisin level has a negative correlation with the TNF-α and IL-6 plasma levels (Li et al., 2017; Figure 3). Irisin consumption reduces the expression of pro-inflammatory cytokines, the expression of monocyte chemoattractant protein-1 (MCP-1), and the migration of macrophages, then induces the phenotypic change of macrophages from pro-inflammatory to anti-inflammatory (Kim and Song, 2018; Figure 4). Irisin plays a role in reducing insulin resistance and glucose homeostasis in AD subjects, and stimulation of fibronectin type III domain-containing protein 5 (FNDC5)/irisin by endurance exercise has been reported. In addition, in brain ischemia, the significant increase of ROS and malondialdehyde is reduced by irisin treatment (Kim and Song, 2018). Therefore, it seems that regular physical exercise can prevent age-related dementia through FNDC5, slow the progression of AD, and improve cognitive function. While the deletion of irisin through the global deletion of FNDC5 leads to cognitive disorders and memory disorders in the elderly (Zhou et al., 2019; Islam et al., 2021; Madhu et al., 2022). In other words, genetic deletion of FNDC5/irisin leads to cognitive dysfunction in exercise, aging, and AD. In mice lacking FNDC5, neurons in the dentate gyrus are morphologically, transcriptionally, and functionally abnormal. Depletion of FNDC5 is also ameliorated by delivery of irisin to the dentate gyrus so that irisin appears to be the active region. Therefore, increasing the delivery of irisin through the liver leads to the enrichment of central irisin and is sufficient to improve cognitive deficits and neurological disorders in AD mouse models (Islam et al., 2021). After 5 weeks, swimming exercise and L-carnosine intake (100 mg/kg/day) normalizes the expression of FNDC5/irisin in the hippocampus and is accompanied by a decrease in Aβ and phosphorylated tau protein. These interventions also improve BDNF expression and insulin sensitivity and reduce cognitive disorders (Hegazy et al., 2022). It is worth noting that telomere length shortening with aging plays a critical role in the pathogenesis of AD through oxidative stress and inflammation and is associated with cognitive impairment, increased Aβ, and tau phosphorylation. While exercise significantly affects DNA damage and telomere length, plasma irisin level significantly correlates with telomere length. Therefore, plasma irisin seems related to telomere length and shows anti-aging properties (Rana et al., 2014; Figure 4).

## Exercises suitable for improving Alzheimer’s disease

To date, there is no consensus on the best method of exercise training to improve AD patients. However, aerobic exercise such as walking is possible for people with AD and is associated with better cognitive function. Strength training can be very important for patients with AD because muscle mass and strength are low in people with AD. In addition, balance exercises improve the standing position of people with AD and reduce the risk of falling in the later stages. In general, multifactorial exercises such as aerobic exercises, strength exercises, and balance and coordination exercises provide important benefits for this population (Paillard et al., 2015). In patients with mild AD, moderate to high-intensity aerobic exercise on a treadmill and stationary bike (70–80% of maximum heart rate, for 1 h, three times a week for 16 weeks) preserves cognitive function and improves physical parameters (Jensen et al., 2019b). Acute aerobic exercise (cycling for 20 min at moderate-intensity with a 60% maximum heart rate) along with cognitive games in people with moderate AD (age 69 years) improves cognitive functions and improves the time of functional activities. Therefore, aerobic exercise along with cognitive games should be promoted in moderate AD (Ben Ayed et al., 2021). In people with AD, walking with conversation has a better effect than walking alone, because the social effect of exercise is important for this population. In addition, walking in sunlight improves sleep in AD patients (Paillard et al., 2015). People with AD (84 years) after a six-month training program (aerobic exercises, resistance exercises, flexibility, and body posture) for 45–55 min in each session, twice a week, improve physical and cognitive functions. Physical functions include cognitive function, chair stand, arm curl, 2-min step, 8-foot up-and-go, chair sit-and-reach, and back scratch tests as well as waist circumference (Sampaio et al., 2019). Patients with mild AD participating in supervised aerobic exercise (60-min sessions three times a week for 16 weeks) increase cognitive function, quality of life, and ability to perform daily activities, and decrease neurological and depressive symptoms (Hoffmann et al., 2016). In elderly people, cycling for 6 months with moderate-intensity (20–50 min per session, 3 times a week) shows that increasing cognitive function and hippocampus volume increases physical performance and quality of life. This training method also curbs the high costs of treating people with dementia (Yu et al., 2014). Resistance exercises (three sets of 20 repetitions in five exercises for 16 weeks) cause a significant improvement in movement at home, climbing stairs, standing on the floor, wearing socks, agility, lower limb strength, balance, and flexibility in patients with AD (Garuffi et al., 2013). In the animal model, running on a treadmill is the most common exercise method. Aerobic exercises are commonly used with a duration of 60 min per session with moderate-intensity, 5 days a week and 4 or 12 weeks. This protocol can significantly reduce the level of Aβ or pro-inflammatory proteins (De Sousa et al., 2021). In mice, short-term resistance training leads to improvement of cognitive function, pathological changes, inhibition of neuroinflammation in the frontal cortex and hippocampus, reduction of Aβ, reduction of tau protein, and also synaptic plasticity. Therefore, resistance training seems to be an alternative strategy to delay the development of AD (Liu et al., 2020). In addition, resistance exercise (4 weeks of intermittent training) for mice with AD causes reduction of Aβ, reduction of local inflammation, memory enhancement, cognitive improvement, and protection of cortical and hippocampus against degeneration. The resistance program also leads to improved behavioral changes, and increased microglial cells in the hippocampus (Campos et al., 2023). Aerobic, resistance and combined training for 6 weeks and 3 times a week on AD shows that the freezing time (lack of movement in the head, trunk, and limbs) is reduced with aerobic training and working memory is improved with all types of training. All exercises increase glutathione levels as an antioxidant, decrease malondialdehyde and increase serum IGF-I levels. Increases in APP mRNA levels are attenuated by compound exercise. Aβ levels are also reduced by resistance training and combined training in the hippocampus and by various types of exercise in the cortical part, while cortical NGF is increased by combined exercise. Finally, various types of exercise seem to have protective effects on AD by reducing oxidative stress, reducing Aβ, and increasing the antioxidant system and brain flexibility (Özbeyli et al., 2017). In general, it seems that different types of moderate-intensity training (3–5 times a week and 1 h per session) can be useful for delaying the onset and treatment of AD.

## Conclusion

Alzheimer’s disease (AD) is a chronic neurological disorder in the elderly with dementia, memory loss, and severe cognitive decline. AD imposes high treatment costs on the individual and healthcare systems. In contrast, Moderate-intensity voluntary exercise (30 min to 1 h per session, 5 days per week, for 4–26 weeks) restores behavioral and psychological symptoms of dementia (BPSD), hippocampal and amygdala memory in Alzheimer’s disease (Lin et al., 2015; Fleiner et al., 2017). This process seems to be through increasing the levels of p-TrkB, p-AKT, and p-PKC and decreasing the level of Aβ, phosphorylation of tau, and APP. Aerobic exercise (for 20 min to 1 h per day, 3–5 days per week, with an intensity of 50–75% of the maximum oxygen consumption, for 12–24 weeks) through increasing synaptic flexibility prevents hippocampal volume reduction, dementia, decreased spatial learning and memory, and loss of synapse function in people with AD (Mu et al., 2022). These exercises induce BDNF binding to TrkB and NGF binding to TrkA to induce cell survival and neuronal plasticity. Therefore, exercise increases brain function by causing structural and neurochemical changes in the hippocampus and temporal lobe that are important for dementia, learning, and memory (Duzel et al., 2016; Matura et al., 2016). After aerobic training and high-intensity interval training (treadmill running for 3–7 weeks every day for 30 min, 5 days a week), increases in VEGF, angiopoietin 1 and 2, NO, tPA, and HCAR1 in cerebral vasculature have been reported. Therefore, exercise causes angiogenesis and increased blood flow in the cerebellum, striatum, motor cortex, and hippocampus, and these effects seem to be important in the treatment of AD-induced dementia (Morland et al., 2017). In the hippocampus, 12 weeks of HIIT and MICT significantly reduce Aβ, mitochondrial fragmentation, DRP1, and FIS1, and improve MFN1, MFN2, OPA1, and mitochondrial morphology. Therefore, exercise improves AD subjects’ exploratory behavior, dementia, spatial learning, and memory ability, possibly by improving mitochondrial morphology and dynamics (Li et al., 2019). Exercise appears to increase mitochondrial activity by activating the SIRT1-FOXO1/3-PINK1-Parkin pathway by increasing the NAD+/NADH ratio, thus contributing to the reduction of mitochondrial dysfunction associated with AD neurodegeneration. In humans, acute exercise as an anti-inflammatory response causes an acute increase of IL-6 and an increase of anti-inflammatory cytokines such as IL-1RA and IL-10. This process further inhibits IL-1β and TNF-α. In animals, moderate to high-intensity training (3–4 months) inhibits inflammatory markers such as IFN-γ, IL-6, CRP, TNF-α, sTNFR1, IL-1β, COX- 2, and NF-kB. Therefore, exercise inhibits the pro-inflammatory M1 phenotype and stimulates the anti-inflammatory M2 phenotype in the brain to restore dementia and BPSD (Choi et al., 2014; Matura et al., 2016; De la Rosa et al., 2020). In the finnish geriatric intervention study to prevent cognitive impairment and disability (FINGER) multi-intervention studies, aerobic exercise during starvation and fasting improves mitochondrial function, oxidative stress, neuroinflammation, neurodegeneration, amyloid beta and tau proteins, memory impairment and cognitive disorders by increasing ketone and glucose absorption. Aerobic exercise significantly increases the plasma levels of BDNF, nerve growth factor, motor activity, cognitive impairment, exploratory behavior, and spatial memory in people with AD (Choi et al., 2018; Wang et al., 2020). Hippocampal neurogenesis through running is positively correlated with increased synaptic plasticity, spatial memory, pattern separation, and decreased dementia and enhances memory performance (Ahlskog et al., 2011; Duzel et al., 2016). Irisin is a myokine that is secreted from skeletal muscles in response to exercise and enters the central nervous system by crossing the BBB and increasing the expression of BDNF to enhance memory and learning and decreased dementia (Hegazy et al., 2022; Madhu et al., 2022). Irisin protects the hippocampus by suppressing Aβ accumulation and promotes hippocampal proliferation through STAT3 signaling. Irisin activates Akt and ERK1/2 signaling pathways in brain tissue and has neuroprotective effects. Thus, combined exercises such as aerobic exercises, strength exercises, and balance and coordination exercises seem along with cognitive and social activities provide important benefits for people with AD-induced dementia (Sampaio et al., 2021).

## Author contributions

The author confirms being the sole contributor of this work and has approved it for publication.
